# N,N-Dimethylglycine in patients with progressive multiple sclerosis: result of a pilot double-blind, placebo, controlled randomized clinical trial

**DOI:** 10.1186/s42466-021-00126-z

**Published:** 2021-05-24

**Authors:** Thomas Wolfsegger, Klaus Böck, Wolfgang Schimetta, Tim J. von Oertzen, Hamid Assar

**Affiliations:** 1 Department of Neurology 1, Kepler University Hospital, Neuromed Campus, Wagner-Jauregg-Weg 15, 4020 Linz, Austria; 2 Department of Applied Systems Research and Statistics, Johannes Kepler University, Linz, Austria

**Keywords:** N,N-Dimethylglycine, Multiple sclerosis, Nutrition therapy, Dietary supplement, Mobility

## Abstract

Oral administration of N,N-Dimethylglycine (DMG), a tertiary amino acid, presumably enhances oxygen utilization by tissue and complex with free radicals. Beneficial effects are improved endurance performance and reduction fatigue in humans and animals. This pilot study reports the results over a one-year double-blind, placebo-controlled trial of DMG in 30 randomized patients with progressive multiple sclerosis. No treatment effects were found between the placebo group and the DMG group for disability, fatigue, cognitive, or gait parameters.

## Introduction

N,N-Dimethylglycine (DMG) has been marketed as a dietary supplement since 1974 and has seen wide use in both humans and animals. Health field research found that DMG may boost physical and mental performance in athletes and animals [1, 2]. Dimethylglycine generates two carbon molecules such as sarcosine glycine serine and ethanolamine’s all of which are beneficial to the cell. For example, glycine function are an important inhibitory neurotransmitter of the central nervous system [3]. It is used to produce phosphocreatine, a high energy phosphate molecule, used in muscle tissue and the tissue of the central nervous system [4].

In general, there is substantial interest in the ability of dietary interventions to influence multiple sclerosis (MS)-related outcomes. To date, there are limited evidence-based recommendations regarding dietary interventions for MS [5].

Based on these experimental data and claims of efficacy, we initiated a double-blind, placebo-controlled, randomized pilot study to assess the efficacy on disability, fatigue, cognitive, and motor performance of orally administered DMG in patients with progressive MS.

## Methods

Ambulatory patients in the age range of 30–60 years with a diagnosis of primary (PPMS, *n* = 8) or secondary progressive (SPMS, *n* = 22) MS [6] were included in this randomized, double-blind, placebo-controlled pilot study*.* Patients were randomized (assigned in a 2:1 ratio) to receive either DMG (at a dose of 125 mg/day) or placebo (Lactose-monohydrate). DMG or placebo was administered orally over 12 months. Fatigue Impact Scale (FSI-D), Paced Auditory Serial Addition Test (PASAT), and gait analysis (shown in Table 1) were assessed at baseline and at months 3, 6, and 12. Expanded Disability Status (EDSS) examination was performed at baseline and 12 months. Adherence to disease-modifying treatments in all patients remained stable during the study period. In all patients, no immunomodulatory therapy was applied during the study period.

**Table 1:** Characteristics of patients

	DMG		Placebo	
	Baseline (*n* = 20)	12 months (*n* = 17)	Baseline (*n* = 10)	12 months (*n* = 9)
Age (years)	54.9 ± 5.7		52.1 ± 5.9	
Sex (M/F)	6/14		1/9	
SPMS (M/F)	4/10		0/8	
PPMS (M/F)	2/4		1/1	
Weight (kg)	75.8 ± 16.3		75.6 ± 21.3	
Height (cm)	167.6 ± 10.1		166.4 ± 7.1	
Disease duration (years)	13.8 ± 9.3		18.8 ± 10.0	
Study variables				
EDSS (score)	4.6 ± 2.0 (5.3)	4.5 ± 2.0 (4.5)	5.2 ± 1.5 (6.0)	5.3 ± 1.7 (6.0)
FIS-D (score)	85.8 ± 26.8 (86.5)	76.0 ± 35.0 (86.0)	73.3 ± 25.7 (73.5)	81.6 ± 21.5 (89.0)
PASAT (score)	40.6 ± 8.8 (39.0)	41.4 ± 12.0 (36.0)	35.6 ± 7.0 (34.0)	44.2 ± 9.7 (42.0)
Gait velocity (km/h)	2.5 ± 1.1	2.5 ± 1.2	2.6 ± 0.7	2.6 ± 0.8
Stride length (m)	0.50 ± 0.1	0.52 ± 0.2	0.49 ± 0.1	0.55 ± 0.2
Stride variability (%)	4.2 ± 2.7	4.4 ± 4.5	12.9 ± 22.9	11.7 ± 17.1
6 min Walking Test (m)	259.9 ± 141.8	267.2 ± 135.6	268.6 ± 80.5	277.3 ± 84.5
Knee angle stance (left/2 + right/2)(°)	16.3 ± 4.9	16.4 ± 4.4	17.8 ± 7.5	17.5 ± 6.1
Knee angle swing (left/2 + right/2)(°)	48.8 ± 10.5	49.1 ± 9.2	47.8 ± 7.7	49.7 ± 6.7

*SPMS* secondary progressive MS, *PPMS* primary progressive MS, *EDSS* Expanded Disability Status Scale, *FIS-D* Fatigue Impact Scale, *PASAT* Paced Auditory Serial Addition Test, *mean ± sd* () = median

Gait parameters (knee motion) were performed with 3D-body markers by the motion capture system SIMI®, Germany. Gait velocity, stride length, and variability were quantified by a mobile insole foot pressure measurement (Medilogic®, Germany).

The primary endpoint was the effect on clinical, cognitive, and gait parameters after 12 months of DMG application (delta 12 months - baseline).

All data sets of continuous variables were checked for normal distribution (Kolmogorov-Smirnov test with Lilliefors significance correction, type I error = 10%). Group comparisons of normally distributed data sets were performed by the t-test for independent samples (test for variance homogeneity: Levene test, type I error = 5%). Data sets of continuous variables without normal distribution were compared by the Mann-Whitney U test. Dichotomous variables were compared by the Fisher’s exact test. The relationship between baseline demographics, concomitant medication, and concomitant disease was analyzed by multiple regression analysis.

## Result

Thirty patients were included for the intention to treat analysis. In the DMG group, 17/20 patients, and in the placebo group 9/10 patients completed the trial. Drop out reasons were lack of efficacy (*n* = 2 DMG) and major protocol violence (*n* = 1 DMG; *n* = 1 placebo group).

In the baseline evaluation, no significant difference could be found between the DMG versus the placebo group.

After 12 months of intervention, no significant group differences were found in clinical assessments for disability status (EDSS score 0.8 points; *p* = 0.558), the impact of fatigue (FIS-D score 5.6 points; *p* = 0.334), and cognitive performance (PASAT score 2.8 points; *p* = 0. 077). Moreover, no significant differences could be evaluated in all gait parameters (6 min walking test 10.1 m; *p* = 0.218, gait velocity 0.1 km/h; *p* = 0.586, stride length 3 cm; *p* = 0.209 and stride variability 7.3%; *p* = 0.656) as well as knee range of motion stance (1.1°; *p* = 0.323) and swing (0.6°; *p* = 0.072) (Table 1).

No significant relationships were found between the study variables, concomitant medications and concomitant disease. Treatment with DMG had a similar safety profile to placebo assessed by self-reported side effects (diary protocol).

## Discussion

This is the first randomized, double-blind, placebo-controlled clinical trial on the efficacy of orally administered DMG in MS. Our results demonstrate that oral DMG (125 mg/day) has no therapeutic effect upon fatigue, cognitive, and gait performance as well as disability status in MS patients. No differences between the study groups were found concerning all evaluated parameters over the study period.

For the first time, Russian studies in the1950s on athletes reported increased oxygen efficiency, reduced fatigue, and increased performance. Beginning in 1975, work was done with DMG in the United States to verify these claims [2].

DMG has been suggested as a beneficial supplement and has been used to combat fatigue, improve metabolism, and strengthen the immune system [1, 2]. On the back of these, some nutritional companies have included DMG in their products and made claims that it has beneficial effects on endurance performance by combating fatigue and enhancing metabolism.

However, there is at present, little evidence to back up these claims. Most research appears to point to there being little or no benefit to DMG supplementation [2]. For example, tested with college athletes, DMG increased maximum oxygen absorption. Athletes taking DMG had a 23.6% increase over placebo control in the length of exercise time before exhaustion [7]. The results of these studies could not be verified in adults and children [8, 9].

In general, research on humans has failed to find any conclusive benefits on measured physiological variables on aerobic performance in healthy subjects with DMG supplementation (intake 100 mg - 200 mg daily) [2, 8–10].

Research studies concerning DMG and MS are not available. A recently published review about other dietary interventions in MS shows inefficient evidence of whether supplementation with antioxidants, omega-3, omega-6, or vitamin D has any impact on MS-related outcomes [5].

Limitations of this pilot trial included imbalances between patient groups and the small sample size. We are unable to assess whether a modification of the intake profile or a different dosage of DMG might be effective. These considerations should be taken into account in any further planning and design of a larger efficacy study.

## Conclusion

In this first pilot study, 12 months of treatment with 125 mg DMG once daily did not improve cognition, fatigue, or motor performance in patients with progressive MS.

## Data Availability

The data generated and analyzed in this pilot study are available from the corresponding author.

## Abbreviations

DMG: N,N-Dimethylglycine
MS: Multiple Sclerosis
FSI-D: Fatigue Impact Scale Paced
PASAT: Auditory Serial Addition Test
EDSS: Expanded Disability Status
SPMS: Secondary progressive MS
PPMS: Primary progressive MS
